# Rapid Self‐Assembly Mini‐Livers Protect Mice Against Severe Hepatectomy‐Induced Liver Failure

**DOI:** 10.1002/advs.202309166

**Published:** 2024-03-17

**Authors:** Miaomiao Luo, Jiahui Lai, Enhua Zhang, Yue Ma, Runbang He, Lina Mao, Bo Deng, Junjin Zhu, Yan Ding, Jialyu Huang, Bin Xue, Qiangsong Wang, Mingming Zhang, Pengyu Huang

**Affiliations:** ^1^ State Key Laboratory of Advanced Medical Materials and Devices, Engineering Research Center of Pulmonary and Critical Care Medicine Technology and Device (Ministry of Education) Institute of Biomedical Engineering Tianjin Institutes of Health Science Chinese Academy of Medical Science & Peking Union Medical College Tianjin 300192 China; ^2^ School of Life Science and Technology ShanghaiTech University Shanghai 201210 China; ^3^ Center for Reproductive Medicine Jiangxi Maternal and Child Health Hospital Jiangxi Branch of National Clinical Research Center for Obstetrics and Gynecology Nanchang Medical College Nanchang 330006 China; ^4^ Core Laboratory Department of Clinical Laboratory Sir Run Run Hospital Nanjing Medical University Nanjing 211166 China

**Keywords:** Acute liver injury, Bioartificial liver, Hepatocyte, Rapid self‐assembly

## Abstract

The construction of bioartificial livers, such as liver organoids, offers significant promise for disease modeling, drug development, and regenerative medicine. However, existing methods for generating liver organoids have limitations, including lengthy and complex processes (taking 6–8 weeks or longer), safety concerns associated with pluripotency, limited functionality of pluripotent stem cell‐derived hepatocytes, and small, highly variable sizes (typically ≈50–500 µm in diameter). Prolonged culture also leads to the formation of necrotic cores, further restricting size and function. In this study, a straightforward and time‐efficient approach is developed for creating rapid self‐assembly mini‐livers (RSALs) within 12 h. Additionally, primary hepatocytes are significantly expanded in vitro for use as seeding cells. RSALs exhibit consistent larger sizes (5.5 mm in diameter), improved cell viability (99%), and enhanced liver functionality. Notably, RSALs are functionally vascularized within 2 weeks post‐transplantation into the mesentery of mice. These authentic hepatocyte‐based RSALs effectively protect mice from 90%‐hepatectomy‐induced liver failure, demonstrating the potential of bioartificial liver‐based therapy.

## Graphical abstract

A straightforward and time‐efficient approach is developed for creating rapid self‐assembly mini‐livers (RSALs), essentially scaled‐up liver organoids, using primary hepatocytes within 12 h. RSALs are functionally vascularized within 2 weeks post‐transplantation into the mesentery of mice and effectively protect mice from 90%‐hepatectomy‐induced liver failure, demonstrating the potential of bioartificial liver‐based therapy.

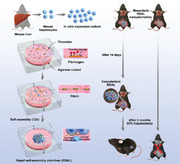

## 1 Introduction

The liver plays a crucial role in carrying out essential physiological functions, including the synthesis of serum proteins, detoxification, metabolism, and excretion.^[^ ^1^ ^]^ Notably, the liver demonstrates robust regenerative capacity, capable of rapidly restoring its original size and functionality within two weeks, even when up to 2/3 of its mass has been surgically removed.^[^ ^2^ ^]^ However, in cases of severe liver failure in patients, this regenerative capacity diminishes, leaving the patient unable to restore liver function, ultimately leading to mortality.^[^ ^3^ ^]^ Advancements in bioartificial liver supporting devices, which include bioreactors housing hepatocytes, have shown significant clinical benefits in restoring lost liver functions among patients suffering from liver failure.^[^ ^4^ ^]^ However, this treatment typically necessitates hospitalization, and patients frequently exhibit limited tolerance for blood purification.^[^ ^4a^ ^]^ Consequently, there is growing interest in the development of transplantable bioartificial liver tissue as a more promising alternative to address these challenges, offering the potential to compensate for impaired liver functions resulting from liver damage.

Numerous efforts have been made in the construction of bioartificial liver for transplantation purposes. Recently, remarkable progress has been made in developing small prototypes known as liver organoids, typically with dimensions ≈100 µm in diameter.^[^ ^5^ ^]^ However, when fabricating bioartificial liver tissue on a larger scale, a host of significant challenges arises. A primary challenge is the acquisition of a sufficient number of safe, reliable, and functional hepatocytes for use as seeding cells. Although substantial research efforts have been dedicated to induced pluripotent stem cells (iPSCs),^[^ ^6^ ^]^ embryonic stem cells (ESCs),^[^ ^7^ ^]^ and transdifferentiated cells,^[^ ^8^ ^]^ persistent concerns related to issues like teratoma formation, genome instability, high costs, time‐intensive and complex processes, and limited functionality continue to hinder progress. In recent years, substantial progress has been made in the in vitro expansion of primary hepatocytes, presenting a more cost‐effective and safer source of hepatic cells.^[^ ^9^ ^]^

A significant challenge arises from the limitations of current technologies in producing larger liver tissue containing a sufficient mass of functional cells capable of compensating for liver damage.  When organoid increases in size, and_ _issues such as necrosis emerge due to the build‐up of toxic metabolic wastes and the depletion of local oxygen and nutrients. The slow growth rate of liver organoids further compounds these problems. Therefore, urgent exploration of strategies for rapidly assembling high‐density hepatocytes is imperative, aiming to minimize the necrotic core and enhance the size of bioartificial livers.

In addressing this challenge, we directed our focus toward fibrin, a naturally occurring polymer critical in the final stage of coagulation. In this complex physiological process, exposure of a wound to air triggers a cascade of responses, including vascular constriction and platelet aggregation. A pivotal step in this sequence is the activation of thrombin, which converts soluble fibrinogen in the blood vessels into insoluble fibrin strands. These fibrin strands form a meshwork or scaffold that captures blood cells, particularly red blood cells and platelets, forming a stable blood clot that arrests further blood loss and promotes wound healing.^[^ ^10^ ^]^ This in vivo coagulation process can be effectively mimicked in vitro, where thrombin converts fibrinogen into fibrin.^[^ ^11^ ^]^ The presence of Arg‐Gly‐Asp (RGD) motifs within the fibrin matrix facilitates cell adhesion via interactions with cell‐integrin receptors, making fibrin an ideal scaffold for enhancing various cellular activities, including adhesion, proliferation, and differentiation.^[^ ^11^ ^]^ Due to its ease of preparation, adjustability, cell support, biocompatibility, and innate biological safety, fibrin has found widespread application in the field of regenerative medicine.^[^ ^11^, ^12^ ^]^ Furthermore, agarose is commonly used for spheroid generation, owing to its property of providing a non‐adherent concave surface that prevents cell attachment to the bottom of the culture dish.^[^ ^13^ ^]^

**Figure 1:**
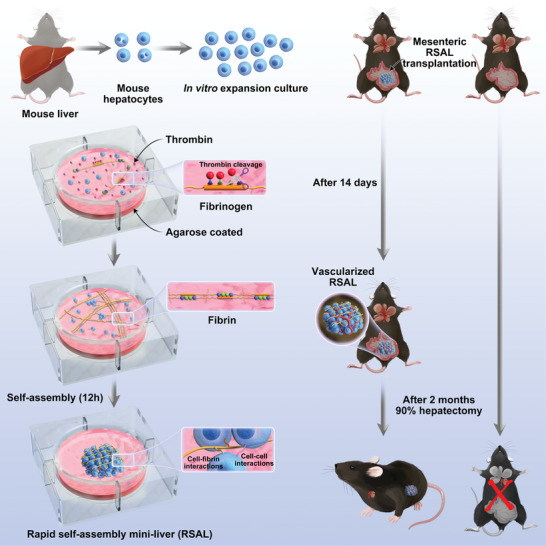
Schematic illustration of the fabrication and application of RSAL.

In this study, we engineered a rapid self‐assembly mini‐liver (RSAL) by utilizing the natural physiological coagulation process through fibrinogen and thrombin. Thrombin added to the cell‐fibrinogen solution initiates the cleavage of fibrinogen into fibrin strands, forming a scaffold for hepatocytes to aggregate. A mini‐liver sphere thus forms in the supports of the concave bottom derived from agarose (**Figure** **1**).

RSAL exhibits several advancements compared to liver organoids, including increased and consistent size (uniform 5.5 mm vs 50–500 µm in diameter ^[^ ^7^ ^]^), enhanced cellular viability (99% viability vs necrotic core^[^ ^14^ ^]^), and a shorter construction process (12 h vs 6–8 weeks or longer^[^ ^15^ ^]^). Moreover, fibrin, being of endogenous origin, demonstrates excellent biocompatibility and undergoes suitable biodegradation. Additionally, RSAL demonstrates elevated expression levels of liver‐specific marker genes in vitro compared to hepatocytes cultured in 2 dimension (2D). Importantly, when surgically implanted into the mesentery of mice, RSALs establish a functional vascular system connected to the host mouse within 14 days post‐transplantation. Mice transplanted with RSALs demonstrate significantly increased survival rates after the removal of 90% of the host liver tissue, highlighting the in vivo functionality of RSALs and their therapeutic potential in the treatment of liver failure.

## 2 Results and Discussion

### 2.1 Fabrication of Rapid Self‐Assembly Mini‐Liver (RSAL)

To obtain a sufficient quantity of functional hepatocytes, we isolated primary hepatocytes from C57BL/6NCrl mice and subsequently expanded them to over 10^8^. This expansion was achieved using a dedicated in vitro expansion culture medium designed to support the extensive proliferation of primary mouse hepatocytes (Figure S1, Supporting Information). Even after 15 passages, the expanded hepatocytes retained their mature liver functions, including glycogen storage and the capability to uptake acetylated low‐density lipoprotein (Figure S2, Supporting Information).

**Figure 2:**
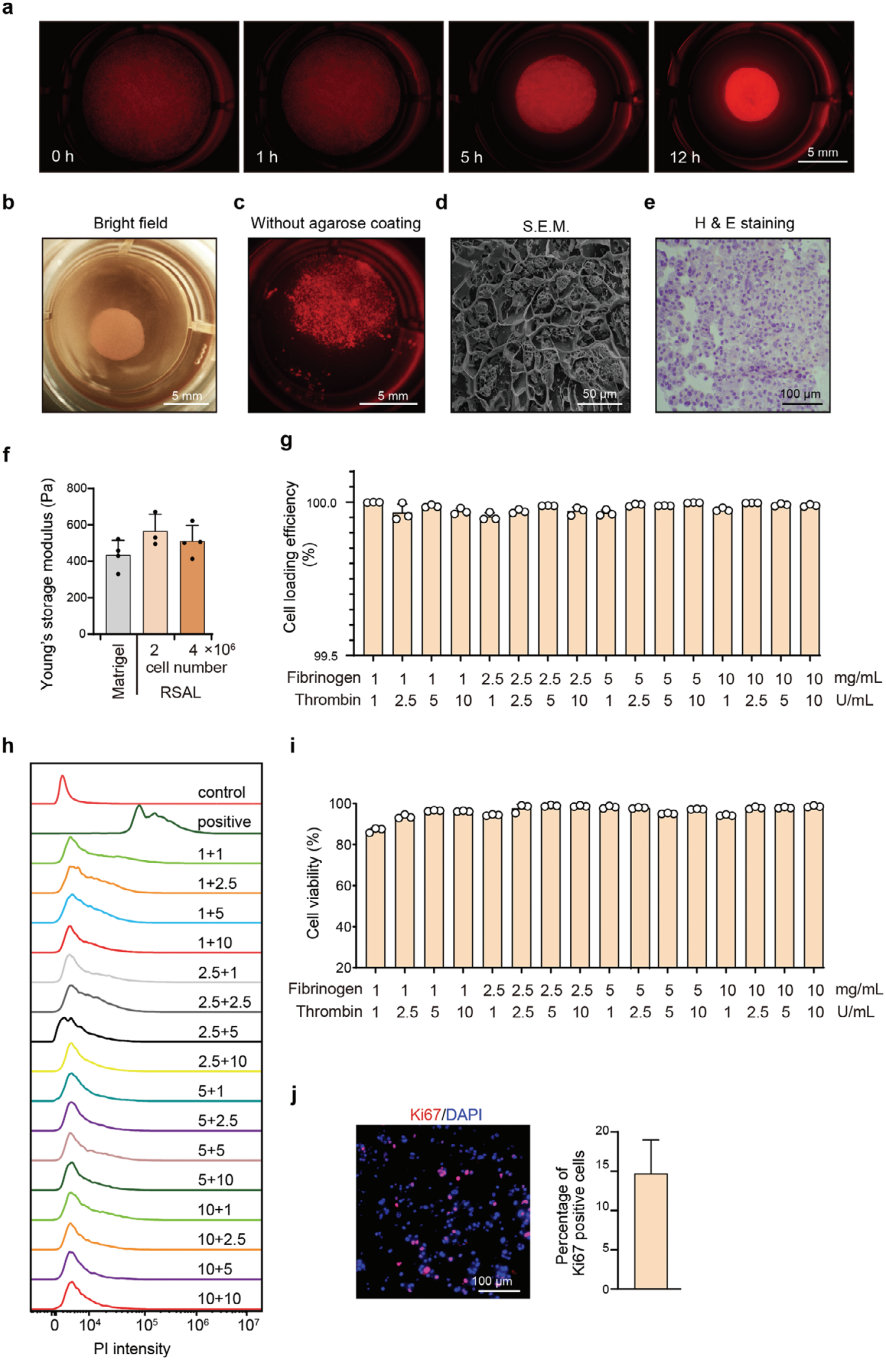
Fabrication of RSAL. a) Time‐lapse fluorescence imaging of RSAL over a 12 h period. b) Bright field image of RSAL. c) Image of RSAL without agarose coating. d) SEM image of RSAL. e) H&E staining of RSAL. f) Young's modulus of Matrigel and RSAL. g) Cell loading efficiency of RSAL constructed by different proportions of fibrinogen and thrombin. h) Flow cytometry histogram profiles of RSAL cell viability constructed by different proportions of fibrinogen and thrombin. i) Quantification of cell viability in RSAL constructed by different proportions of fibrinogen and thrombin. j) Immunofluorescence staining of Ki67 in RSAL and quantification analysis of the percentage of Ki67 positive cells.

In the construction of RSAL, a fibrinogen solution containing hepatocytes was rapidly mixed with a thrombin solution and placed on an agarose‐coated plate. Remarkably, despite being cultivated in a 2D environment, the hepatocytes demonstrated an inherent capacity to self‐organize into a 3D bioartificial liver within just 12 h after the addition of thrombin (**Figure** **2**a,b). These RSALs exhibited robust mechanical stability, making them easily amenable to physical manipulation (data not shown). RSALs generated from 2 × 10^6^ hepatocytes in 100 µL fibrinogen solution reached a diameter of 5.5 mm (Figure S3, Supporting Information). More importantly, the average diameter of RSALs constructed with 1 × 10^6^ hepatocytes in 50 µL fibrinogen solution was 2.6 mm (Figure S3, Supporting Information). These results indicated that RSAL size is directly proportional to the cell count, facilitating consistent sizes, a crucial aspect for RSAL standardization. In contrast, liver organoids typically vary in size, ranging from 20 to 200 µm in diameter.^[^ ^7^ ^]^ More importantly, this system also could be utilized for self‐assembling various other types of cells. For instance, MEF cells could self‐assemble into a mini sphere within only 12 h (Figure S4, Supporting Information).

We then investigated the required amounts of fibrinogen and thrombin for the self‐assembly of hepatocytes. It was observed that hepatocytes failed to aggregate when exposed to low concentrations of fibrinogen and thrombin. However, when the concentrations of fibrinogen and thrombin exceeded 1 mg mL^−1^ and 1 U mL^−1^, respectively, hepatocytes reliably self‐assembled into a sphere. Interestingly, the specific proportion of fibrinogen and thrombin appeared to have little effect on the formation of RSAL (Figure S5, Supporting Information). Besides, the diameter of RSAL formed by the 10 mg ml^−1^ fibrinogen in Figure S5 (Supporting Information), is generally larger than other columns. it is not due to the different pore sizes formed with varying concentrations of fibrinogen. Because fibrin hydrogels with different fibrinogen concentrations exhibit similar pore sizes (Figure S6, Supporting Information). We posit that the fibrin hydrogel with a higher fibrinogen concentration possesses more solid content in the RSAL, consequently leading to a larger RSAL diameter.

The concurrent presence of agarose and hepatocytes is essential for the formation of RSAL. First, without agarose coated at the bottom of the culture plate, hepatocytes adhere to the plate's surface and fail to self‐assemble, even when exposed to high concentrations of fibrinogen and thrombin (Figure 2c). In contrast, when the plate was coated with agarose, hepatocytes rapidly self‐assembled into a macroscopically visible 3D mini‐liver sphere (Figure 1A; Figure S7, Supporting Information). This effect can be attributed to agarose's ability to prevent cellular adhesion to the substrate and promote cell aggregation by creating a concave and low adhesion bottom. Besides, in the absence of hepatocytes, fibrinogen could not self‐assemble into a 3D structure. Instead, we observed the formation of a transparent thin film hydrogel constructed by fibrin after the addition of thrombin (data not shown), underscoring the pivotal role of hepatocytes in driving the self‐assembly of fibrin into a 3D structure.

Subsequently, scanning electron microscopy (SEM) and hematoxylin and eosin (H&E) staining were conducted, revealing that the hepatocytes established intrinsic cell junctions within RSALs (Figure 2d,e). Importantly, similar pore sizes were observed between the RSALs and the thin film formed by fibrin in the absence of hepatocytes (Figure 2d; Figure S8, Supporting Information). This suggests that the inclusion of hepatocytes has minimal impact on the inherent pore sizes of the hydrogel formed by fibrin. These findings demonstrate that the hepatocytes are capable of establishing strong connections with each other to generate an integrated miniature tissue within just 24 h.

Moreover, RSALs constructed with varying volumes consistently exhibited similar tight connections among mouse hepatocytes, indicating their potential applicability across a range of sizes (Figure S9, Supporting Information). In comparison, organoids require 6–8 weeks or more to achieve tight connections between cells.^[^ ^16^ ^]^

The mechanical properties of RSALs were evaluated by measuring their Young's storage modulus. The results revealed that the Young's storage modulus of RSAL exhibited negligible changes when the cell density within the RSAL ranged from 2 million to 4 million (Figure 2f). This observation indicates that hepatocytes within the range of 2 to 4 million cells do not exert a significant influence on the mechanical strength of RSAL. Interestingly, a noteworthy convergence of Young's modulus values between RSAL and Matrigel was observed due to Matrigel's mechanical strength being conducive to cell growth,^[^ ^17^ ^]^ suggesting the suitability of RSAL's mechanical strength for hepatocyte growth.

To optimize RSALs, we conducted a comprehensive investigation into the effects of varying quantities and proportions of fibrinogen and thrombin during their formation. Remarkably, the construction of RSALs with 2 × 10^6^ hepatocytes resulted in an impressive high cell loading efficiency of 99.5%, achieving a cell density of up to 1.6 × 10^5^ cells mm^−3^. During our exploration of fibrinogen and thrombin concentrations ranging from 1 to 10 mg mL^−1^ and 1 to 10 U mL^−1^, respectively, we observed no significant effects on cell loading efficiency based on the quantity and ratio of fibrinogen and thrombin (Figure 2g).

Additionally, it is noteworthy that in vitro expanded hepatocytes demonstrated excellent tolerance to the self‐assembly process under various amounts and proportions of fibrinogen and thrombin. The cell viability remained consistently above 85% and could reach as high as 99% after 24 h of self‐assembly, with minimal cell death observed (Figure 2h,i). This remarkable survival rate can be attributed to the utilization of intrinsic fibrinogen, secreted by hepatocytes in vivo, as the biomaterial. It creates a hepatocyte‐friendly microenvironment that supports the 3D organization of hepatocytes. Furthermore, in vitro expansion culture of hepatocytes further acclimated them to the in vitro environments. For consistency, in the subsequent experiments, we used 10 mg mL^−1^ of fibrinogen and 10 U mL^−1^ of thrombin for the construction of RSALs unless specified otherwise.

Notably, 13.9 ± 4.0% of the hepatocytes displayed positive staining for Ki67 after 24 h of self‐assembly, indicating ongoing cell proliferation within RSALs (Figure 2j). Thus, RSALs provide a conducive microenvironment that supports both the survival and continuous proliferation of hepatocytes.

### 2.2 RSALs Maintain Liver Functions In Vitro

**Figure 3:**
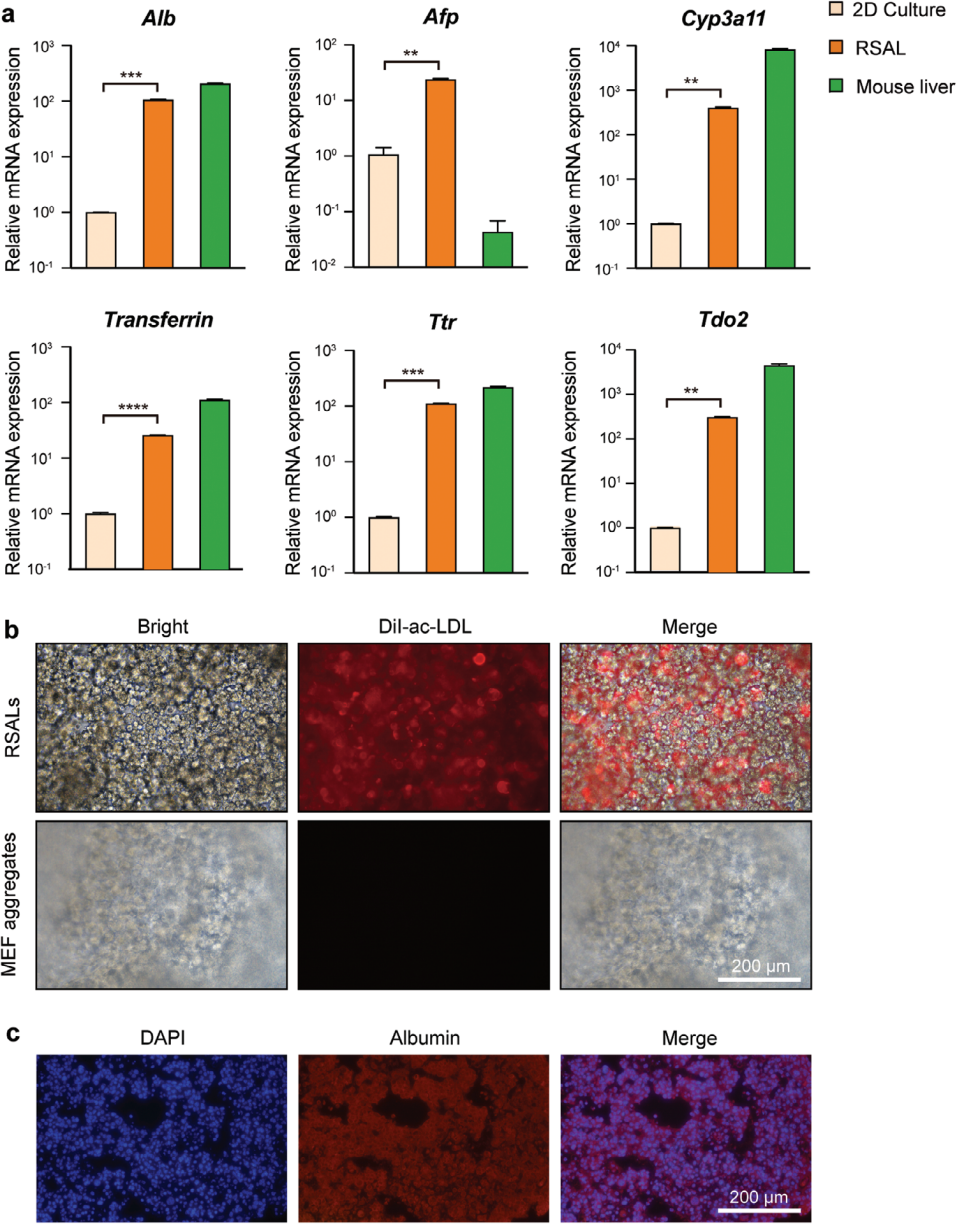
RSALs maintain liver functions in vitro. a) Quantitative PCR analysis of expression of the hepatic marker gene in RSAL after 10 days of in vitro culture. Results are presented as mean ± s.d., n = 3. b) DiI‐ac‐LDL uptake in RSAL (red). The negative control, established through the rapid self‐assembly of mouse embryonic fibroblasts (MEF), exhibited an absence of red fluorescence. c) Immunofluorescence staining of mouse albumin in RSAL. The unpaired student's *t*‐test was utilized for comparisons between the groups. ^*^ *p *< 0.05, ^**^ *p *< 0.01, ^***^ *p *< 0.001, ^****^ *p *< 0.0001.

After successfully constructing RSALs, we evaluated their liver functions in vitro. Initially, we analyzed the expression of liver‐specific genes using quantitative PCR. Compared to hepatocytes cultured in a 2D environment, RSALs exhibited either increased or comparable expression levels of liver function‐related genes, including albumin (ALB), transferrin, transthyretin (TTR), tryptophan 2,3‐dioxygenase (Tdo2), and alpha‐fetoprotein (AFP) (**Figure** **3**a). Remarkably, RSALs displayed significantly higher expression of *Cyp3a11*, which encodes the drug metabolism enzyme cytochrome P450 3A11, compared to hepatocytes in 2D culture. These results suggest that hepatocytes in RSALs possess enhanced drug metabolism capability. These findings are consistent with previous reports, indicating that hepatocytes undergo further maturation and acquire increased liver functions in 3D culture compared to 2D culture.^[^ ^7^, ^18^ ^]^

Furthermore, we observed that RSALs retained the ability to uptake DiI‐conjugated acetylated low‐density lipoprotein (DiI‐ac‐LDL) (Figure 3b), a commonly used method for assessing low‐density lipoprotein metabolism in hepatocytes.^[^ ^19^ ^]^ These observations further support the notion that hepatocytes can maintain their liver functions within the RSALs. Additionally, we performed immunofluorescence staining of albumin in RSALs, which demonstrated the expression of albumin, a liver‐specific protein (Figure 3c), consistent with the gene expression of *Alb* in RSALs as shown in Figure 3a.

### 2.3 Neovascularization and Long‐Term Sustenance of RSALs In Vivo

**Figure 4:**
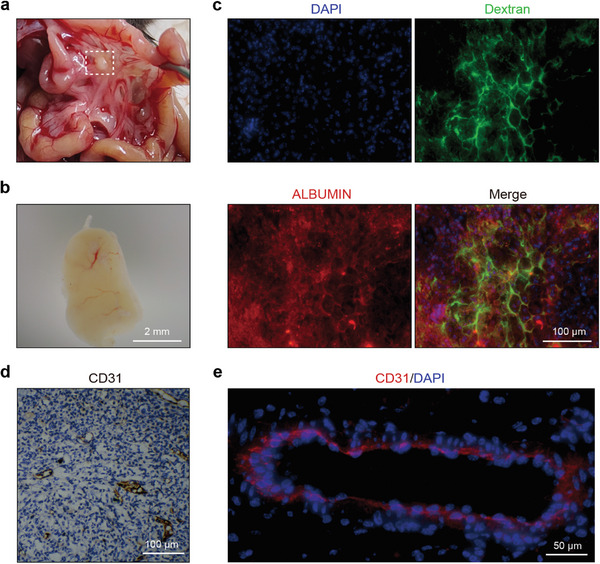
Neovascularization and long‐term sustenance of RSALs in vivo. a,b) Macroscopic observation of RSAL 14 days after transplantation. c) Infusion of dextran to display the formation of functional vessels and red fluorescence to indicate the expression of albumin. d) Immunohistochemistry staining of mouse CD31 in RSAL. e) Immunofluorescence staining of mouse CD31 in RSAL.

Blood vessels played a critical role in artificial liver construction. They transport oxygen and nutrients to cells, and remove metabolic by‐products and waste from cells, therefore ensuring the viability, and functionality of the artificial liver. Besides, the artificial liver generated an ectopic liver by establishing connections with the body's blood vessels. After successfully constructing relatively large RSALs, we transplanted them into the mesentery of C57BL/6NCrl mice to investigate their in vivo therapeutic potential. Following 14 days of transplantation, the RSAL remained intact within the mouse's mesentery, demonstrating its capability for long‐term sustenance in vivo (**Figure** **4**a). Interestingly, we observed further compaction of the RSAL post‐transplantation. Remarkably, neovascularization of the RSAL occurred, with observable blood flow within the RSAL (Figure 4b; Figure S10, Supporting Information). Additionally, we injected fluorescein‐conjugated dextran into the mice and observed the perfusion of dextran into the vascular system of the RSAL, indicating a functional connection between the vascular system of RSAL and the host mice (Figure 4c).

As shown in Figure 4c, the neovascular network intertwined with ALBUMIN‐positive hepatocytes within the RSAL plays a critical role in facilitating the transport of oxygen, nutrients, and metabolic waste. To further validate the development of the vascular system, we conducted CD31 staining on RSAL in14 days post‐transplantation, revealing a clearly formed vascular system marked by CD31‐positive endothelial cells (Figure 4d,e). Moreover, we co‐stained CD31 with a hepatocyte‐specific protein FAH to further elucidate the spatial relationship between blood vessels and the positive cell population in Figure S11 (Supporting Information). The figure illustrated that CD31‐positive blood vessels were situated among Fah‐positive cells, suggesting that blood vessels may facilitate the transport of oxygen and nutrients to hepatocytes while aiding in the removal of metabolic waste products produced by hepatocytes, thus promoting a long‐term survival of transplanted hepatocytes. Besides, a 20‐day in vitro culture of RSAL was performed to illustrate its fate without neovascularization. After being cultured for 20 days in vitro, we observed significant necrosis at the core of the RSAL (Figure S12, Supporting Information), a phenomenon commonly observed in conditions with insufficient oxygen and nutrients. In contrast, the neovascularization of RSAL after transplantation facilitated the transport of oxygen and nutrients, allowing for the prolonged survival of hepatocytes within RSAL and enhancing their hepatic functionality. Therefore, these findings substantiate that RSALs are capable of neovascularization after transplantation, supporting their sustained viability in vivo over an extended period.

### 2.4 RSAL Protected Mice from Liver Failure

**Figure 5:**
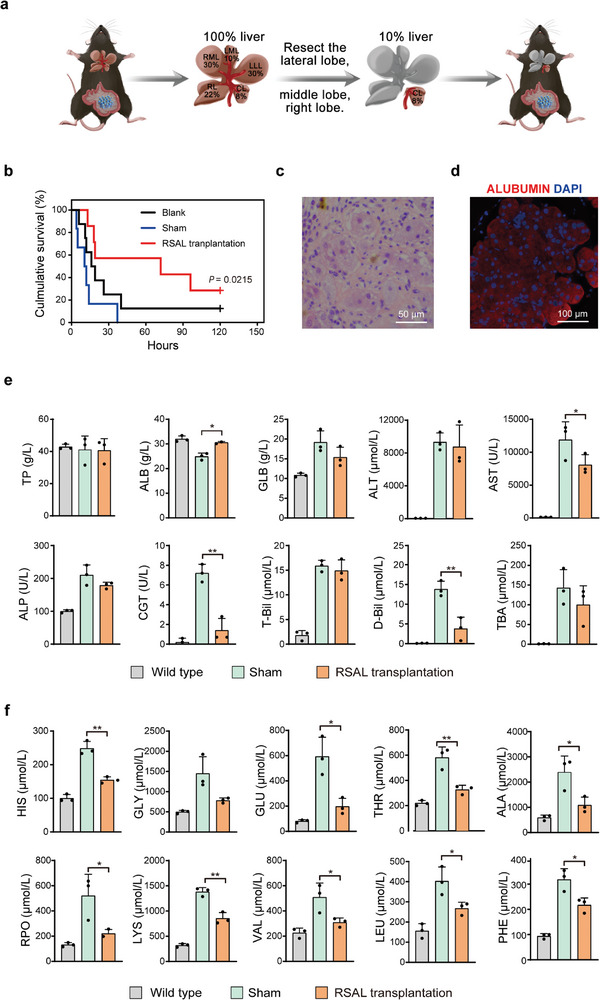
RSAL transplantation rescues 90%‐hepatectomy mice. a) Schematic illustration of 90%‐hepatectomy. b) Survival curve of wild type C57BL/6NCrl mice (n = 9), sham‐operated C57BL/6NCrl mice (n = 7), and RSAL transplantation C57BL/6NCrl mice (n = 7) suffering from 90%‐hepatectomy. Curves were analyzed 2 months after transplantation. In this study, four RSALs were transplanted into the mesentery of each mouse. c) HE staining of RSAL within C57BL/6NCrl mice survived up to day 5 after 90% hepatectomy. d) Immunofluorescence staining of ALBUMIN in RSAL. e) Serum levels of total protein, albumin, globulin, alanine aminotransferase, aspartate aminotransferase, glutamic‐pyruvic transaminase, alkaline phosphatase, gamma‐glutamyl transpeptidase, total bilirubin, direct bilirubin, and total bile acids in wild type C57BL/6NCrl mice, sham‐operated C57BL/6NCrl mice, and RSAL transplantation C57BL/6NCrl mice 2 days after 90%‐hepatectomy. f) Serum levels of amino acids in wild type C57BL/6NCrl mice, sham‐operated C57BL/6NCrl mice, and RSAL transplantation C57BL/6NCrl mice 2 days after 90%‐hepatectomy. The unpaired student's *t*‐test was utilized for comparisons between the groups. ^*^ *p *< 0.05, ^**^ *p *< 0.01.

As the cell mass of RSALs remains slower than that of the host liver, we transplanted four RSALs into the mesentery of each mouse to investigate their in vivo functions. After 2 months of transplantation, we performed a 90%‐hepatectomy on the host liver to model end‐stage liver failure. This procedure involved surgically excising the lateral lobe, middle lobe, and right lobe, leaving only the caudal lobe, which constitutes ≈10% of the original liver mass (**Figure** **5**a). Among the mice in the blank group (those not undergoing any operation) and the sham group (those receiving sham surgery), 8 out of 9 and all 7 mice, respectively, succumbed within 40 h after the 90%‐hepatectomy (Figure 5b). Remarkably, mice harboring RSALs exhibited a significantly extended lifespan (Figure 5b). Following the 90%‐hepatectomy procedure, RSALs were carefully excised from the mesentery in surviving mice up to day 5. H&E staining results indicated the presence of hepatocytes in the RSAL, suggesting that these hepatocytes could sustain long‐term residence within the body (Figure 5c). Moreover, the RSAL also maintained the expression of ALBUMIN after 60 days’ transplantation (Figure 5d). Besides, the analysis of RSAL sections after 60 days of transplantation revealed no Ki67+ cells in the slices, indicating that hepatocytes ceased proliferation in vivo after 60 days of transplantation (Figure S13, Supporting Information).

We employed serum levels of liver function‐related parameters to assess the in vivo functions of RSAL following 90%‐hepatectomy. These parameters must be maintained within a normal range, as deviations in either excess or deficiency may serve as indicators of abnormal hepatic function in mice. As illustrated in Figure 5e, a noteworthy increase in serum ALB concentrations compared to the sham‐operated group was observed, indicating enhanced liver function in mice bearing RSALs. Additionally, reductions in the serum levels of globulin (GLB), alanine transaminase (ALT), aspartate transaminase (AST), alkaline phosphatase (ALP), gamma‐glutamyl transferase (GGT), total bilirubin (T‐Bil), direct bilirubin (D‐Bil), and total bile acids (TBA) in the RSAL‐transplanted group compared to the sham‐operated group were noted, signifying a substantial reduction in damage to the host liver. These findings correlated with the significantly improved survival of RSAL‐transplanted mice (Figure 5b).

Additionally, we conducted an analysis of serum amino acid concentrations in the mice to further assess the in vivo functions of RSAL. Elevated amino acid levels beyond the normative range can serve as a marker for impaired liver function. As depicted in Figure 5f, mice undergoing RSAL transplantation exhibited a substantial reduction in the levels of amino acids, including histidine, glutamic acid, threonine, alanine, proline, lysine, valine, leucine, and phenylalanine, in contrast to the levels observed in mice subjected to sham surgery. This reduction indicates the restoration of amino acid metabolism capability.

To assess the in vivo toxicity of RSAL, we conducted a blood routine test (Figure S14, Supporting Information). Notably, no discernible changes were observed in any of the groups, underscoring the excellent biocompatibility of RSAL which is a pivotal aspect of their potential in vivo biomedical applications. Furthermore, post the 90%‐hepatectomy, we monitored the body weight of all groups for one month (Figure S15, Supporting Information). The results revealed a transient decrease in body weight during the initial 2 weeks in both the sham and RSAL groups, followed by a rapid return to normal levels over the subsequent month. These findings provide confirmation that RSAL transplantation was well tolerated by the mice.

## 3 Conclusion

In summary, we have successfully engineered RSAL with in vivo functionality by leveraging the intrinsic thrombin‐based physiological coagulation process and utilizing agarose as the supporting substrate. RSAL stands out due to its significantly increased size (≈5.5 mm in diameter), augmented cellular density (1.6 × 10^5^ cells mm^−3^), remarkably swift construction process (completed in 12 h), and an impressive cell survival rate (up to 99%). Our transplantation experiments serve as a proof‐of‐concept, demonstrating that RSAL could function as an in vivo liver support system, effectively compensating for lost liver functions during liver damage. Moreover, the rapid self‐assembly method employed for hepatocytes holds promise for extending its application to assemble various cell types, thereby opening avenues for its use in regenerative medicine and drug screening.

## 4 Experimental Section

### Cell Isolation

Hepatocytes were isolated from male C57 mice aged 6–8 weeks. All animal experiments received approval from the Committee of Institute of Biomedical Engineering, Chinese Academy of Medical Science & Peking Union Medical. The approval number for animal experiments is 22JCZXJC00200 (Beijing‐Tianjin‐Hebei Basic Research Cooperation Special Project).

The procedure involved initial perfusion of the mouse liver with EBSS/EGTA solution lacking Ca^2+^ and Mg^2+^ through the portal vein until complete blood removal. Subsequently, perfusion with 100 mL of EBSS/Hepes solution containing Ca^2+^ and Mg^2+^ followed. This was succeeded by perfusion with 0.025% Type IV collagenase (yuanpei) until the liver tissue achieved a specific flowability. The liver was then excised, and placed in a 6 cm dish, and DMEM (Gibco) containing 10% FBS was added. Gentle mashing of the liver using a yellow pipette tip released the cells, with the removal of the liver capsule. The resulting cell suspension underwent centrifugation at 1000 rpm for 5 min. The pellet was then subjected to further centrifugation at 1000 rpm for 5 min in a 45% Percoll solution to obtain purified mouse hepatocytes. Large cell aggregates were removed using a sterile cell strainer nylon mesh (70 microns).

Mouse embryonic fibroblasts (MEFs) were isolated from 14.5‐day‐old embryos of C57BL/6NCrl mice. The procedure involved washing the mouse embryos three times with PBS, mincing them with scissors, and digesting them with 0.1% Type I collagenase (yuanpei). MEFs were collected by centrifugation at 1000 rpm for 5 min.

### Cell Culture

Following perfusion, primary mouse hepatocytes were seeded onto a dish pre‐coated with collagen type I (Sigma) and initially cultured in DMEM (Gibco) supplemented with 10% FBS, and 5% penicillin G and streptomycin (Gibco) for a duration of 6 h. Subsequently, the culture medium was substituted with hepatocyte expansion medium (Starcell, Shanghai Weien Biotechnology Co., Ltd.) for continued cultivation.

MEFs were cultured in DMEM/F12 supplemented with 10% FBS, 5% penicillin G and streptomycin, 1% nonessential amino acid solution (NEAA) (Gibco), and 10 ng mL^−1^ b‐fibroblast growth factor (b‐FGF) (Peprotech). Cell incubation took place in a 5% CO_2_ incubator at 37 °C, and cells were dissociated using 0.05% trypsin (Gibco) for collection when reaching ≈80% confluence. The culture medium was refreshed every 2 days.

### Retroviral Transduction

For retroviral transduction, a PlemirR plasmid carrying the red fluorescent protein (RFP) gene, along with packaging plasmid psPAX2 and envelop plasmid pMD2.G, were introduced into 293FT cells to generate viral particles. After a 2‐day incubation period, the culture supernatants from infected 293FT cells were filtered through a 0.45 µm filter membrane to remove cell debris. Subsequently, mouse hepatocytes were immediately infected with the produced retroviruses, named MH‐RFP.

### Preparation and Characterization of RSAL

Before assembling the artificial liver, the wells of a 24‐well plate were coated with 300 µL of 1% (w/v) agarose (Sigma) dissolved in DMEM using high‐pressure steam sterilization. Next, two million hepatocytes or MEFs were suspended in 10 mg mL^−1^ of bovine fibrinogen solution (Absin). Then, 100 µL of the fibrinogen‐cell solution was quickly mixed with a 1 mL thrombin (10 U mL^−1^) (Absin) solution and added to the agarose‐coated 24‐well plate. Both fibrinogen and thrombin were dissolved in the culture medium. Following a 12 h incubation period, the artificial liver rapidly self‐assembled.

To observe the formation process of the artificial liver, mouse hepatocytes were infected with retroviruses expressing the RFP gene. Images were captured using a stereomicroscope (Olympus) at 0, 1, 5, and 12 h. SEM images were acquired using a Cryo‐SEM (FEI Quanta 450). The Young's modulus values of the artificial liver and Matrigel (Corning) were measured using nanoindentation techniques.

### Loading Cell Efficiency

Fibrinogen and thrombin were utilized at varying concentrations to fabricate distinct artificial livers following the methodology outlined in Section 2.4. Subsequently, after 24 h of cultivation, the number of MH‐RFP cells leaking into the culture medium were quantified using flow cytometry (FACS). The loading cell efficiency was then calculated using the formula: (MH‐RFP cells leaked in culture medium/total MH‐RFP cells) × 100%.

### Cell Viability

To assess the influence of the self‐assembling process on cell survival, cell viability was analyzed in different artificial livers. Mouse hepatocytes were detached from various artificial livers using 0.1% Type IV collagenase at 37 °C. Subsequently, the hepatocytes were incubated in PI solution for 20 min at 37 °C, shielded from light. Following incubation, the hepatocytes were washed with PBS (yuanpei) and the number of dead cells was determined using FACS. Cell viability in artificial livers was calculated using the formula: (total cells – dead cells/total cells) × 100%.

### Assays for ac‐LDL and ICG Staining

For ac‐LDL staining, artificial livers were treated with 5 µg mL^−1^ Dil‐ac‐LDL (Yeasen) for 1 h at 37 °C. The samples were then washed three times with PBS before observation under a fluorescence microscope.

For ICG staining, artificial livers were incubated with 1 mg mL^−1^ ICG solution dissolved in DMSO for 1 h at 37 °C. Subsequently, the samples were washed three times with PBS before observation under a microscope.

### Real‐Time Quantitative Polymerase Chain Reaction (qPCR)

Total RNA was extracted from artificial livers and MEFs using Trizol according to the manufacturer's protocol. Subsequently, 1 µg of total RNA was reverse transcribed into cDNA using the HiScript Ill 1st Strand cDNA Synthesis Kit (Vazyme), and qPCR was performed on an ABI 7500 fast real‐time PCR system using the Tag Pro Universal SYBR qPCR Master Mix (Vazyme) according to the manufacturer's protocol. The primers used for the qPCR analysis are listed in Table S1 (Supporting Information).

### Transplantation

Male C57BL/6NCrl mice aged 8–10 weeks were anesthetized with 5% chloral hydrate until unconsciousness was achieved. Four of the artificially generated RSALs were then transplanted into the mesentery of each recipient mouse. The mice were allowed to recover in a temperature‐controlled cage and were subsequently returned to their home cages. Post‐transplantation, the mice were intraperitoneally administered dexamethasone (Dex) (MCE) at a dosage of 10 mg kg^−1^ dissolved in a mixture of 3% DMSO, 45% PEG‐300, and 52% saline for the next 3 days. The body weight of mice in different groups was monitored every 5 days post‐transplantation.

### Vessel Imaging

Fluorescein isothiocyanate‐conjugated dextran (2 000 000 MW; Sigma) (1%) was administered into the mice via the tail vein 14 days post‐transplantation of the artificial liver. After 5 min, the mice was anesthetized and the artificial liver was excised. The excised liver was fixed in 4% paraformaldehyde (FPA), dehydrated, paraffin‐embedded, and sectioned into 5 µm slices. Finally, angiogenesis was observed using a fluorescence microscope.

### In Vivo Liver Function Analysis

Two months post‐transplantation, a 90%‐hepatectomy was performed on the mice, precisely resecting the lateral lobe, middle lobe, and right lobe. Blood samples were collected from the surviving mice in different groups and centrifuged them at 4 °C at 3000 rpm for 15 min. The serum was stored at −80 °C before analysis. Total protein (TP), ALB, GLB, ALT, AST, glutamic‐pyruvic transaminase, ALP, GGT, T‐Bil, D‐Bil, and TBA in serum was measured using an automatic biochemical analyzer (Minday).

### Amino Acid Detection

The serum obtained from the aforementioned method was thawed at a controlled temperature of 4 °C and thoroughly vortex mixed. The serum was separated using a waters ACQUITY UPLC I‐CLASS ultra‐high‐performance liquid chromatography system and mass spectrometry analysis was conducted using the Waters XEVO TQ‐S tandem quadrupole mass spectrometry system. Positive ionization was achieved with an applied source voltage of 1.5 kV and a cone voltage of 20 V. The desolvation temperature was set at 600 °C, with a desolvation gas flow rate of 1000 Lh^−1^. The cone gas flow rate was maintained at 10 L h^−1^. Finally, MassLynx quantitative software was used to determine peak areas, with a retention time tolerance of 15 s, and amino acids were quantified using the standard curve method.

### HE Staining and Immunofluorescence Staining

The artificial liver generated in vitro and dissected in vivo was fixed in 4% FPA, dehydrated, paraffin‐embedded, and sectioned into 5 µm slices. The slices were stained according to the manufacturer's protocol for the HE staining kit (Solarbio). For immunofluorescence staining, the slices were hydrated with xylene, gradient ethanol, and water, followed by antigen retrieval. Subsequently, the slices were blocked with 3% BSA for 30 min, incubated them with the corresponding primary antibody diluted in 3% BSA at 4 °C overnight, rinsed them three times with PBS, incubated the slides with an appropriate fluorescent secondary antibody for 1 h at room temperature, and stained the nuclei with DAPI.

### Blood Routine Test

Prior to 90%‐hepatectomy, blood samples from all groups were collected in anticoagulant tubes and stored at 4 °C. Subsequently, they were analyzed using an automatic blood cell analyzer (Shenzhen Mindray Corporation, BC‐5100).

### Statistical Analysis

All data are presented as mean ± standard deviation (s.d.), and each experiment was conducted at least three times. The unpaired student's *t*‐test was utilized for comparisons between two groups, and the log‐rank (Mantel–Cox) test was employed for survival curve analysis. Graphs and statistical calculations were generated using Prism 8 (GraphPad).

## Conflict of Interest

The authors declare no conflict of interest.

## Author Contributions

M.J. and J.L. contributed equally to this work. P.H. proposed the project. P.H., M.Z., and M.L. designed the project. M.L. and J.L. performed most of the experiments and analyzed the data. M.L., J.L., R.H., and E.Z. expanded the hepatocytes. M.L., J.L., Y.M., B.D., J.Z., and Y.D. performed the in vivo transplantation experiments and analyzed the data. L.M., J.H., Q.W, and B.X. provided technical support for the primary cell culture. P.H., M.Z., and M.L. wrote the manuscript. All authors critically revised and approved the manuscript.

## Supporting information

Supporting Information

## Data Availability

The data that support the findings of this study are openly available in No at https://doi.org/[doi], reference number 36.
