# Supplementary material for: Rapid Self‐Assembly Mini‐Livers Protect Mice Against Severe Hepatectomy‐Induced Liver Failure
Source: Adv Sci (Weinh). 2024 Mar 17;11(21):2309166. doi: 10.1002/advs.202309166 (PMC11151005; doi:10.1002/advs.202309166)
Supplement: Supplementary file 1 — Supporting Information [file ADVS-11-2309166-s001.pdf]

ADVANCED SCIENCE
Open Access

Supporting Information

for *Adv. Sci.*, DOI 10.1002/advs.202309166

Rapid Self-Assembly Mini-Livers Protect Mice Against Severe Hepatectomy-Induced Liver Failure

Miaomiao Luo, Jiahui Lai, Enhua Zhang, Yue Ma, Runbang He, Lina Mao, Bo Deng, Junjin Zhu, Yan Ding, Jialyu Huang, Bin Xue, Qiangsong Wang, Mingming Zhang and Pengyu Huang**

Supplementary Information

Rapid Self-Assembly Mini-livers Protect Mice Against Severe Hepatectomy-Induced Liver Failure

Miaomiao Luo[1,], Jiahui Lai[1,], Enhua Zhang[1], Yue Ma[1], Runbang He[1,], Lina Mao[1,], Bo Deng[1,], Junjin Zhu1[1,2], Yan Ding[2,], Jialyu Huang[3], Bin Xue[4], Qiangsong Wang[1], Mingming Zhang[1,*], Pengyu Huang[1, *]

[1]State Key Laboratory of Advanced Medical Materials and Devices, Engineering Research Center of Pulmonary and Critical Care Medicine Technology and Device (Ministry of Education), Institute of Biomedical Engineering, Tianjin Institutes of Health Science, Chinese Academy of Medical Science & Peking Union Medical College, Tianjin, 300192, China

[2]School of Life Science and Technology, ShanghaiTech University, Shanghai, 201210, China

[3]Center for Reproductive Medicine, Jiangxi Maternal and Child Health Hospital, Jiangxi Branch of National Clinical Research Center for Obstetrics and Gynecology, Nanchang Medical College, Nanchang, 330006, China

[4]Core Laboratory, Department of Clinical Laboratory， Sir Run Run Hospital, Nanjing Medical University, Nanjing, 211166, China

*Correspondence: huangpengyu@yeah.net (P.H.),

zhangmm@bme.pumc.edu.cn (M.Z.)

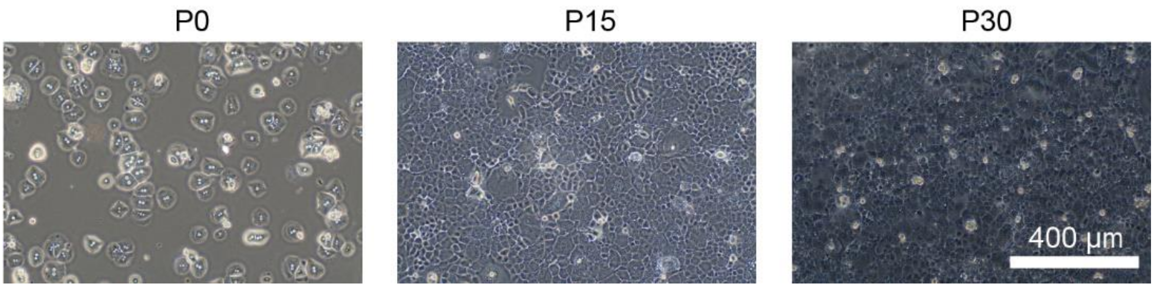

Figure S1. Bright field photograph of mouse hepatocytes in different passages.

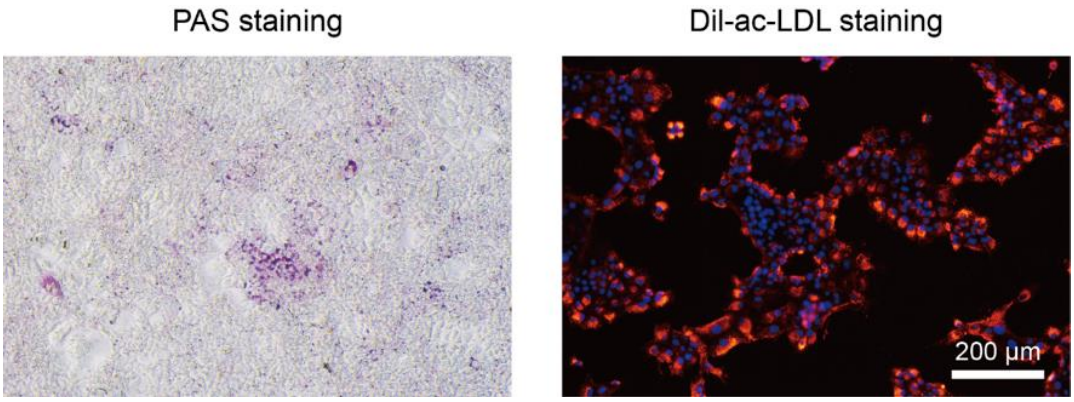

Figure S2. Liver functions of expanded mouse hepatocytes (Passage 15. PAS staining and Dil-ac-LDL staining).

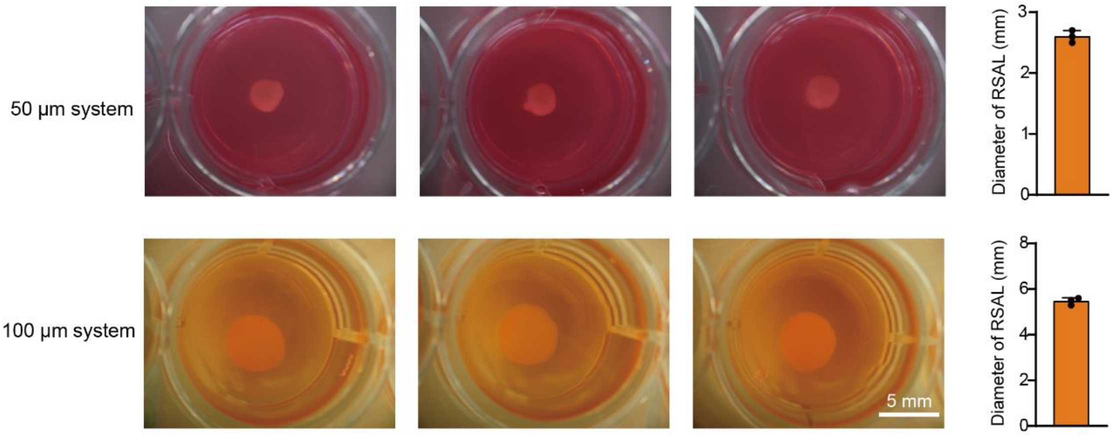

Figure S3. Bright image of RSALs constructed with varying volumes and its average diameter.

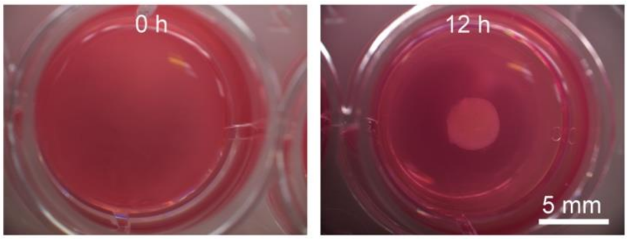

Figure S4. Bright image of rapid self-assembly of MEF in 0 h and 12 h.

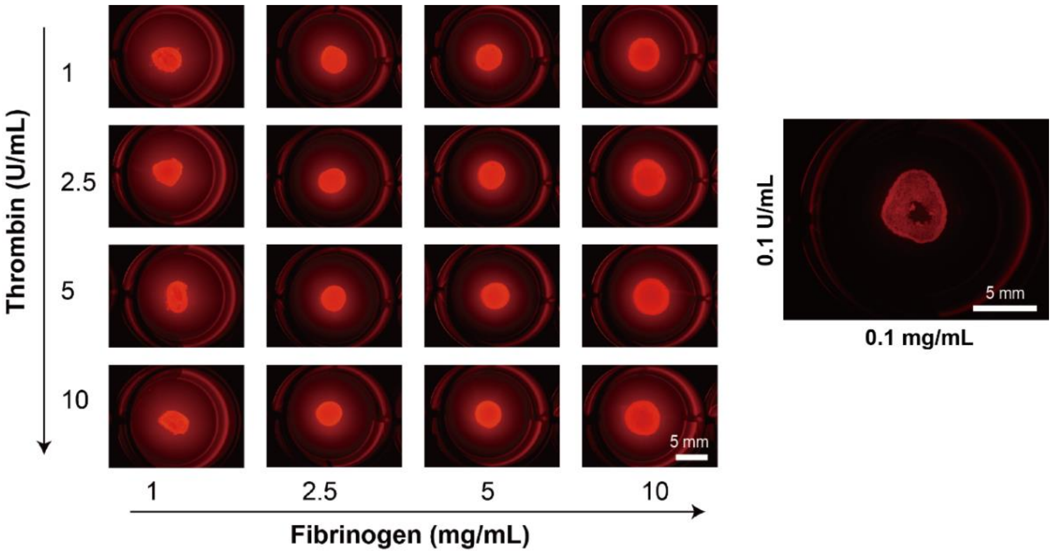

Figure S5. RSALs were assembled using varying proportions of fibrinogen (0.1-10 mg/mL) and thrombin (0.1-10 U/mL).

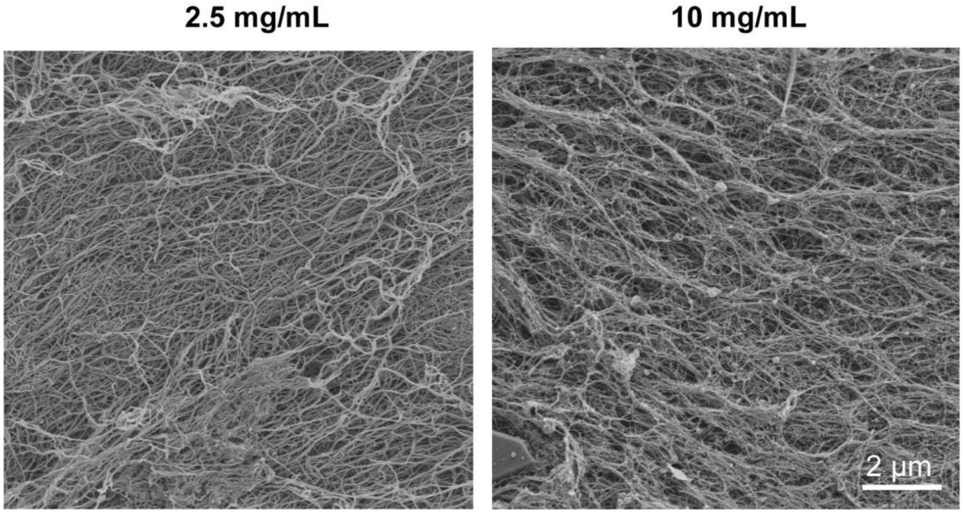

Figure S6. SEM of fibrin hydrogel formed with different concentrations of fibrinogen.

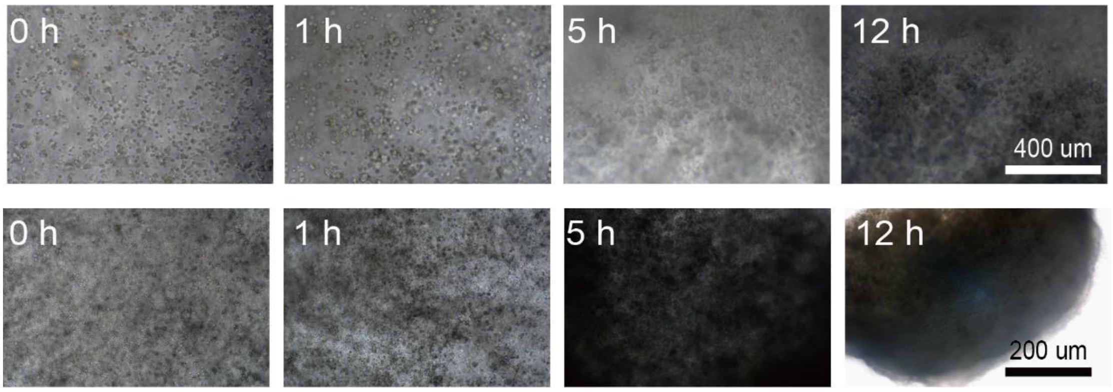

Figure S7. The time-lapse bright field photographs of RSAL exhibited that mouse hepatocytes rapidly self-assemble. The upper and lower images were captured under different magnifications levels with the microscope.

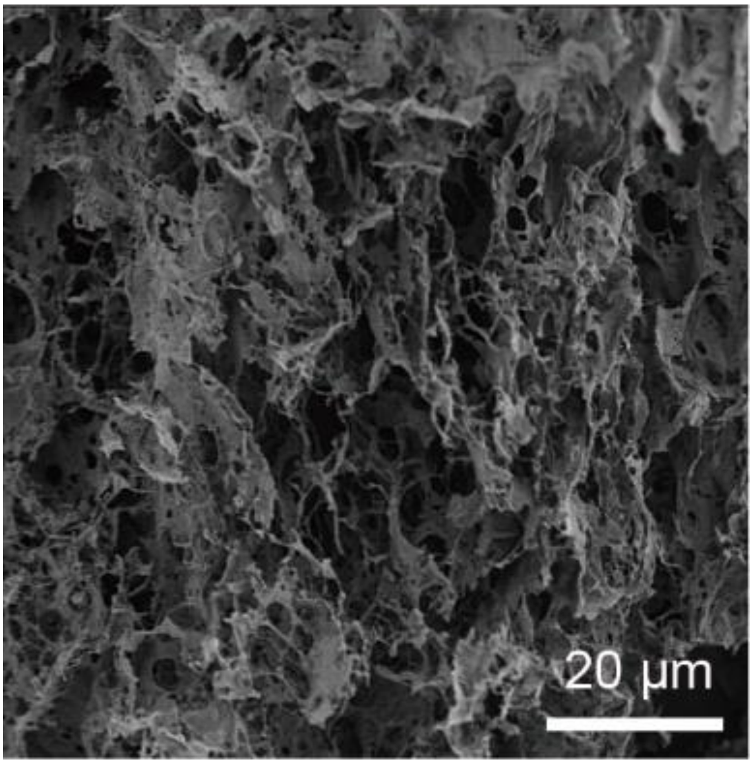

Figure S8. SEM image of fibrin.

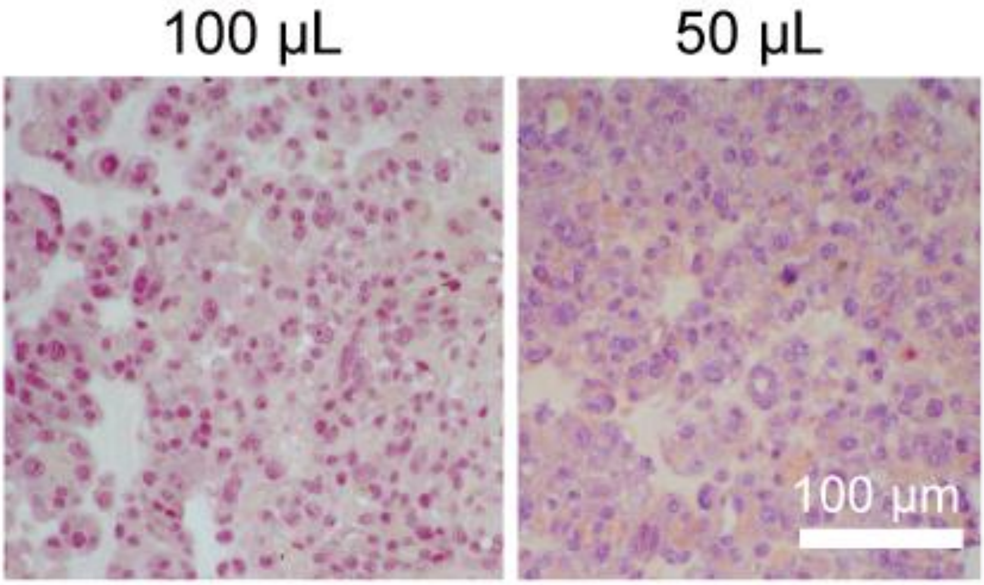

Figure S9. H&E staining of RSALs constructed with varying volumes.

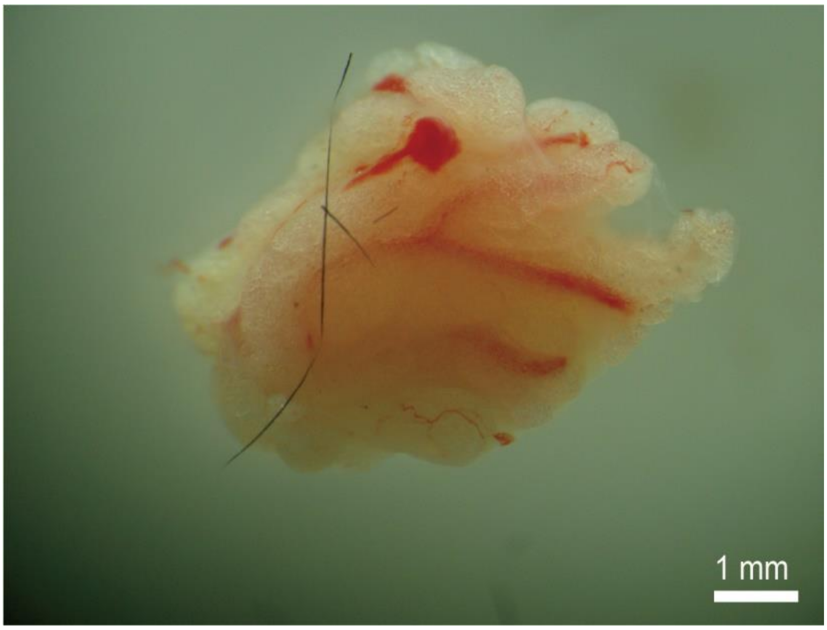

Figure S10. Macroscopic observation of RSAL at 4 weeks post-transplantation.

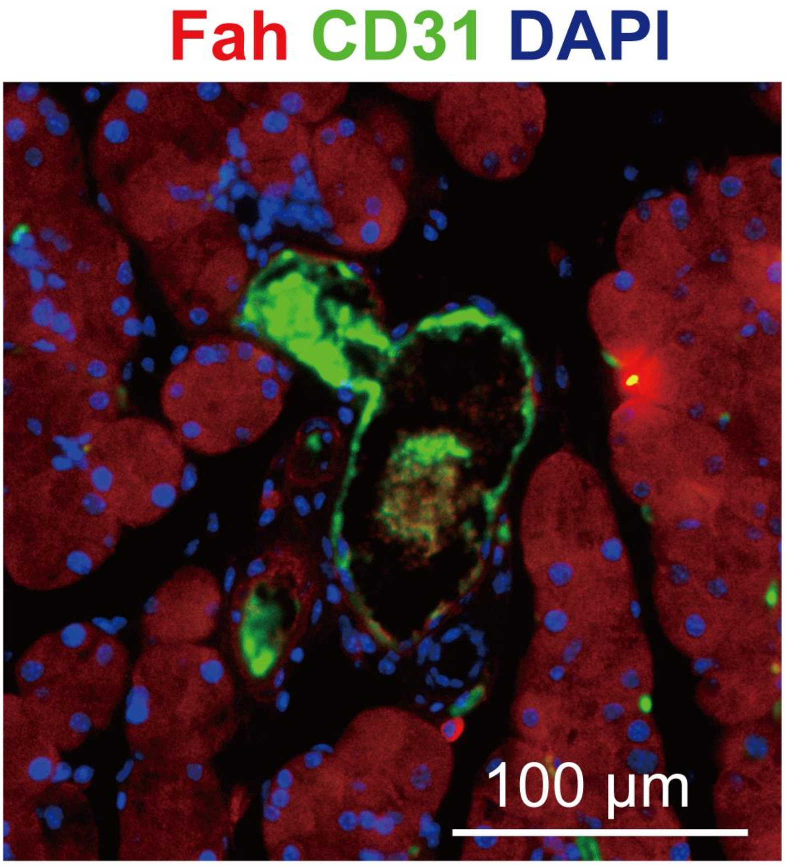

Figure S11. Immunofluorescence staining of co-stain Fah and CD31 in RSAL.

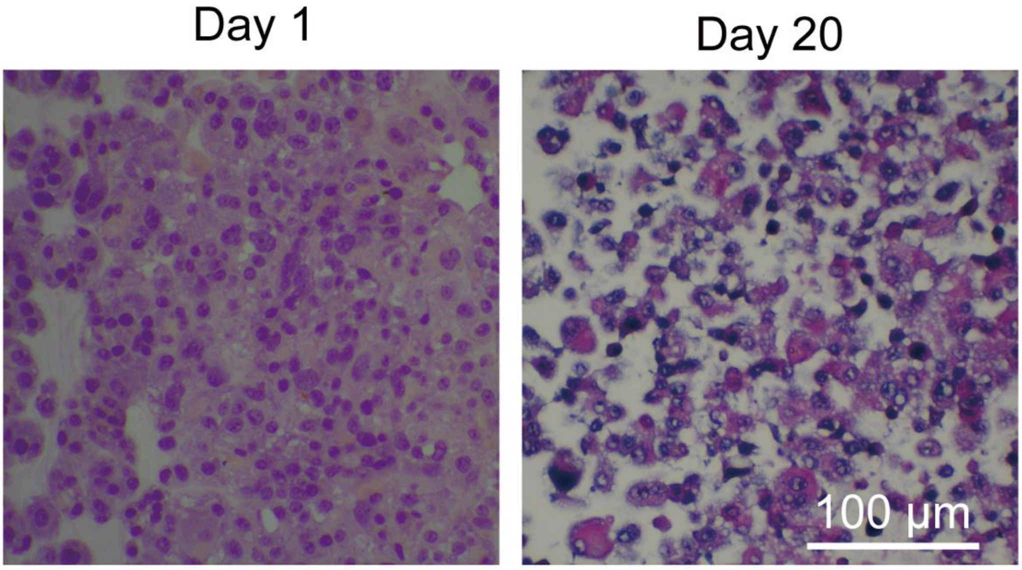

Figure S12. HE staining of RSAL after cultured 1 day and 20 days *in vitro*.

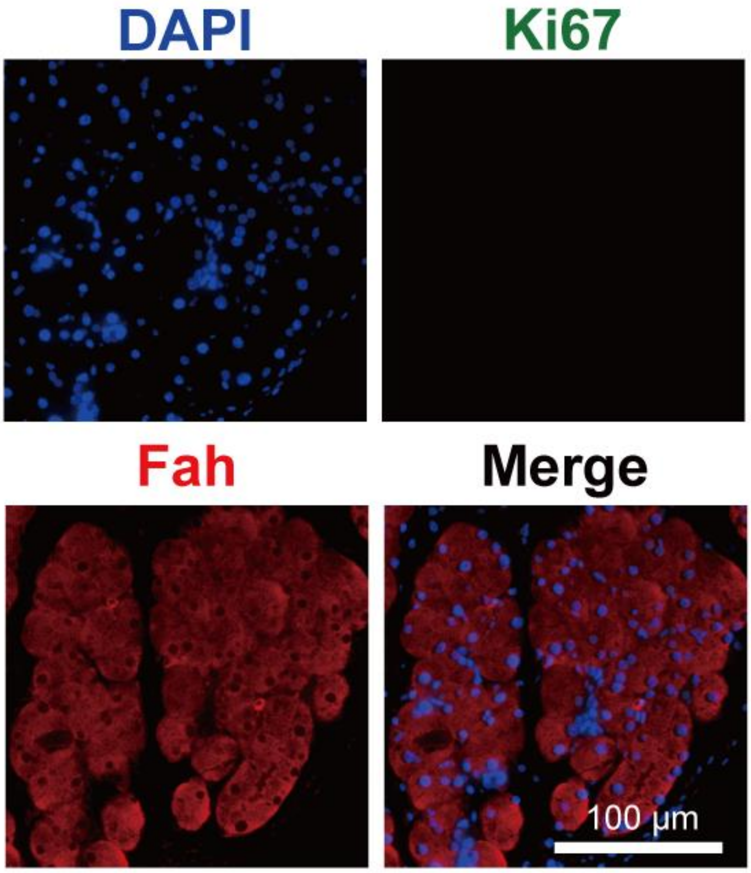

Figure S13. Immunofluorescence staining of Ki67 in RSAL after 60 days post-transplantation.

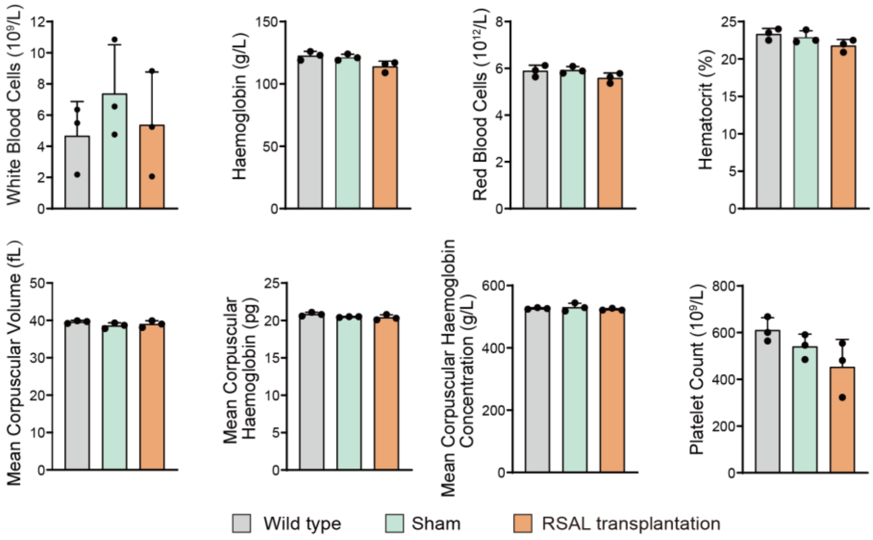

Figure S14. Blood routine value in wild type C57BL/6NCrl mice, sham-operated C57BL/6NCrl mice and RSAL transplantation C57BL/6NCrl mice before 90% hepatectomy.

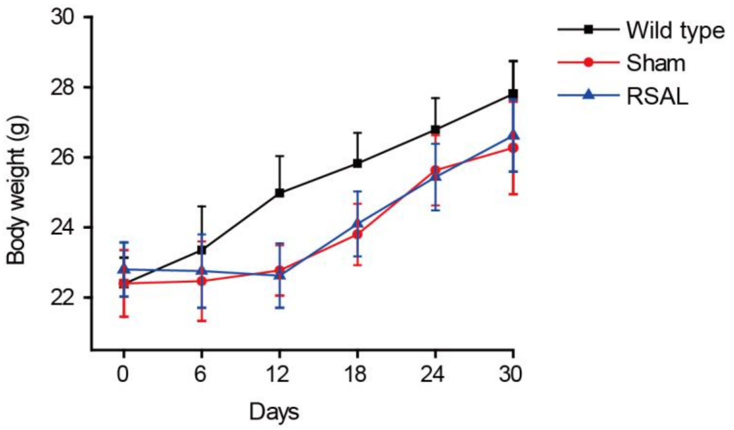

Figure S15. Body weight was monitored every 6 days after RSAL transplantation, n = 8.

Table S1. Primer for Quantitative PCR.

Gene	Forward (5'-3')	Reverse (5'-3')
Alb	CGCCAACTGTGACAAATCCC	GCCTTTCAAATGGTGGCAGG
Afp	CTTCCCTCATCCTCCTGCTAC	ACAAACTGGGTAAAGGTGATGG
CYP3a11	AACCTGGGTGCTCCTAGCAAT	ACCATCAAACAACCCCCATGT
Transferrin	AAGTGCATCAGCTTCCGTGA	TTACCACAGCCACAGCGTAG
Tdo2	ATGAGTGGGTGCCCGTTTG	GGCTCTGTTTACACCAGTTTGAG
Ttr	TTGCCTCGCTGGACTGGTA	TTACAGCCACGTCTACAGCAG
β-Actin	GGCTGTATTCCCCTCCATCG	CCAGTTGGTAACAATGCCATGT
